# Enhancing Protein–Ligand Binding Affinity Predictions Using Neural Network Potentials

**DOI:** 10.1021/acs.jcim.3c02031

**Published:** 2024-02-20

**Authors:** Francesc Sabanés Zariquiey, Raimondas Galvelis, Emilio Gallicchio, John D. Chodera, Thomas E. Markland, Gianni De Fabritiis

**Affiliations:** † Computational Science Laboratory, 16770 Universitat Pompeu Fabra, Barcelona Biomedical Research Park (PRBB), C Dr. Aiguader 88, 08003 Barcelona, Spain; ‡ Acellera Labs, C Dr Trueta 183, 08005 Barcelona, Spain; § Department of Chemistry, Graduate Center, Brooklyn College, 2037 City University of New York, New York, New York 11210, United States; ∥ Computational and Systems Biology Program, Sloan Kettering Institute, Memorial Sloan Kettering Cancer Center, New York, New York 10065, United States; ⊥ Department of Chemistry, 6429 Stanford University, 337 Campus Drive, Stanford, California 94305, United States; # Institució Catalana de Recerca i Estudis Avançats (ICREA), Passeig Lluis Companys 23, 08010 Barcelona, Spain

## Abstract

This letter gives results on improving protein–ligand binding affinity predictions based on molecular dynamics simulations using machine learning potentials with a hybrid neural network potential and molecular mechanics methodology (NNP/MM). We compute relative binding free energies with the Alchemical Transfer Method and validate its performance against established benchmarks and find significant enhancements compared with conventional MM force fields like GAFF2.

## Graphical abstract

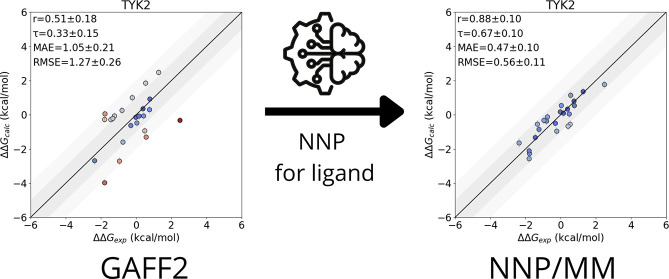

## 1 Introduction

In modern drug discovery, alchemical free energy calculations have emerged as highly efficient tools. Relative binding free energy calculations are widely employed in hit-to-lead approaches, and several commercial and free tools with comparable performance have been developed over the years. However, the accuracy of binding free energy calculations is influenced by the choice of the ligand force field. Most conventional force fields like GAFF,^1,2^ GenFF,^3,4^ and OPLS^5^ often rely on fixed charge molecular mechanics (MM). This lack of important energetic contributions limits their chemical accuracy and leads to poor modeling of torsions.^6–8^

To address these limitations, one approach involves using quantum mechanical (QM) levels of theory to model the ligands while treating the remaining environment with an MM force field in a hybrid potential.^9^ However, QM/MM calculations are significantly more computationally expensive than MM calculations, posing challenges for drug discovery settings, where relative binding free energies (RBFE) calculations may be required for dozens or even hundreds of ligands. Recently, neural network potentials (NNPs) have shown success in predicting QM energies with significantly reduced computational costs compared to QM methods. Notably, the ANI-2x^10^ model supports molecular systems comprising elements H, C, N, O, S, F, and Cl. Moreover, a hybrid method that integrates NNPs and MM, known as NNP/MM^11^, has been developed, offering the potential to model ligands more accurately in RBFE calculations than traditional MM force fields. The Alchemical Transfer Method (ATM) is a recently developed methodology for alchemical free energy calculations that we recently validated that allows an easy implementation of NNPs.^12^ In previous publications, this methodology with MM force fields on a robust data set obtained similar results to other state-of-the-art methods such as FEP+.^13,14^ In this work, we exploit the capabilities of ATM to test the hybrid approach of using ANI-2x^10^ as the neural network potential. Rufa et al. previously managed to reduce the error of absolute binding free energies from 0.97 to 0.47 kcal/mol for a congeneric ligand series for tyrosine kinase TYK2 by correcting the conventional MM simulation with an NNP/MM approach.^15^ ANI-2x has several limitations in terms of not supporting charged molecules and certain elements, but it is otherwise a useful test potential. Our main objective is to test the applicability of this methodology with different ligand force fields and to evaluate the feasibility of an NNP/MM approach in relative binding free energy calculations.

## 2 Methods

In this study, we evaluated a series of targets from both Wang et al.’s^16^ and Schindler et al.’s data sets.^17^ Due to the limitations of ANI-2x,^10^ the NNP of our choice in this study, there is a series of targets from the aforementioned data sets that cannot be computed due to the properties of its ligands. Consequently, we evaluated the following targets: Cyclin-dependent kinase 2 (CDK2), c-Jun N-terminal kinase 1 (JNK1), tyrosine kinase 2 (TYK2), P38 MAP kinase (P38), hypoxia-inducible transcription factor 2 (HIF2A), PFKFB3, spleen tyrosine kinase (SYK), and tankyrase 2 (TNKS2), totaling 301 ligand pairs. For the selected targets, most of the ligands are compatible with ANI-2x, and the rest (and its corresponding ligand pair calculations) were removed from the data set. Due to the higher computational costs related to the integration of NNP into these calculations, a subset of all the possible ligand pairs to be evaluated was selected at random. The workflow in this project is similar to our previous work.^13^ Protein and ligand structures were readily available from Wang’s^16^ and Schindler’s^17^ data sets. Ligands were parametrized with GAFF 2.11^1,2^. The topologies were generated using the *parameterize* ^18^ tool. In contrast to our previous work, we now prepared complex systems using HTMD,^19^ which automated and streamlined the preparation of multiple ligand pairs, along with the automatic selection of binding site residues. However, manual selection of atom indexes for ligand alignment remained necessary. The energy minimization, thermalization, and equilibration steps followed the procedures described in our previous work.^13^ Additionally, the system was annealed to the symmetrical alchemical intermediate (λ = 1/2) for 250 ps. The classical RBFE simulations (GAFF2) were run in triplicate for each ligand pair running an ensemble of 60 ns per replica. Concurrently, we performed the same calculations by using an NNP/MM approach.^11^ This hybrid method allowed us to simulate a portion of the molecular system (the small molecule) with an NNP, while the rest was simulated with MM, providing the ligands with optimized intramolecular interactions. For both approaches, we used the Amber ff14SB parameters^20,21^ as well as the TIP3P water model. Classical RBFE simulations were run at a 4 fs time step while the NNP/MM runs were computed at 1 fs time step, both with the ATM integrator plugin.^22^ Hamiltonian replica exchange along the λ space for each ATM leg was performed with the ASyncRE software,^23^ specially customized for OpenMM and ATM.^24^ Consistent with our previous work, we computed the binding free energies and their corresponding uncertainties from the perturbation energy samples using the Unbinned Weighted Histogram Analysis Method (UWHAM).^25^ The resulting relative binding free energies (ΔΔ*G*) were compared to experimental measurements in terms of the mean absolute error (MAE), root-mean-square error (RMSE), and Kendall Tau correlation coefficient. For all the possible systems, absolute Δ*G* values were computed with cinnabar, an analysis tool to compute absolute binding free energies from ΔΔ*G* values via a maximum likelihood estimator.^26^ Cinnabar also generates the correlation plots and calculates the necessary error and correlation statistics. We compared the obtained values from calculations and the works by Wang et al.^16^ and Schindler et al.^17^ with FEP+. To perform the calculations, we utilized the OpenMM-ML and NNPOps libraries on our in-house cluster, comprising NVIDIA RTX 2080 Ti and NVIDIA RTX 4090 cards. Standard MM calculations were run on GPUGRID. The parallel replica exchange molecular dynamics simulations were conducted by using the OpenMM 7.7 MD engine and the ATM Meta Force plugin, utilizing the CUDA platform.

## 3 Results

The results of our simulations are displayed in Table 1 and Figures 1 and 2 which highlight the relative (Kendall’s rank order correlation) and absolute performances (MAE and RMSE) of the evaluated methods. We do not report the Pearson correlation as well because the value is not significant for (ΔΔ*G*) values as it varies with the choice of the pairs.^27^ We cannot calculate Δ*G* for all pairs since we ran a subset of the original data sets. The computation of Δ*G* for all ligands was not possible due to a poor connection of the perturbation network. Figures S4–S8 display the Δ*G* values and related statistics for the systems that were possible to compute. The NNP/MM method demonstrated superior performance over pure MM runs in both relative and absolute measures. We observe that NNP/MM shows a better correlation coefficient and MAE for all of the evaluated systems but PFKFB3 when compared to ATM with GAFF2 as a force field. In comparison to FEP+, NNP/MM has a lower correlation for two systems (P38 and PFKFB3) and higher MAE for four of them (P38, HIF2A, PFKFB3, and TNKS2). Furthermore, the amount of ligands that are more accurately predicted is increased. In comparison to our GAFF2 runs, MM/NNP predicts a higher percentage of ligands with a MAE lower than both 1 and 1.5 kcal/mol (Table S1). Additionally, there are no major differences between the conformers generated with GAFF2 and ANI-2x as force-fields (Figure S9). We observe for some specific cases how ligands that participated in poor predictions with GAFF2 (MAE > 2 kcal/mol) are now predicted correctly (MAE < 1 kcal/mol) (Table S2). However, this improvement comes at a cost, as NNP/MM calculations are slower than conventional MM calculations.^11^ For instance, an RTX 4090 could yield up to 27 ns/day, whereas an ATM conventional run for a P38 system with 49,000 atoms is able to compute 211 ns/day (Figure S10). This decrease on speed mainly arises due to the limitation of a 1 fs time step with the current ATM integrator. While there is a considerable increase in computational cost for NNP/MM runs, both approaches could benefit from further optimizations. We also evaluate if different timesteps could influence the accuracy of RBFE calculations. We compared the results of the GAFF2 calculations performed in this work with a 4 fs time step with the calculated points from our previous benchmark, that were run at a 2 fs time step (Figure S11). We do not observe any considerable accuracy difference between the calculations at both timesteps. In terms of convergence, we observed that 60 ns per calculation tends to be sufficient. Convergence analysis over time shows good convergence for most cases, as illustrated in Figure S12.

**Figure 1:**
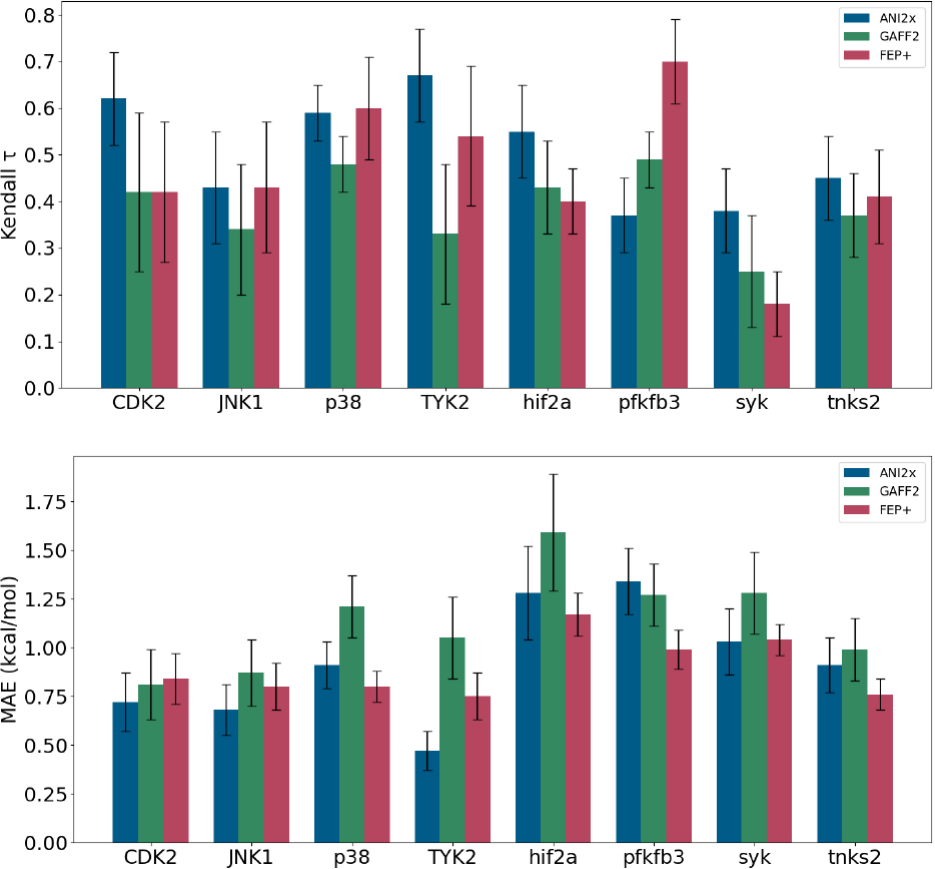
(Top) Kendall tau and (bottom) mean absolute error (MAE) for the ΔΔ*G*s of each protein–ligand system calculated in combination with different force fields and reported estimates using FEP+.^16^

**Figure 2:**
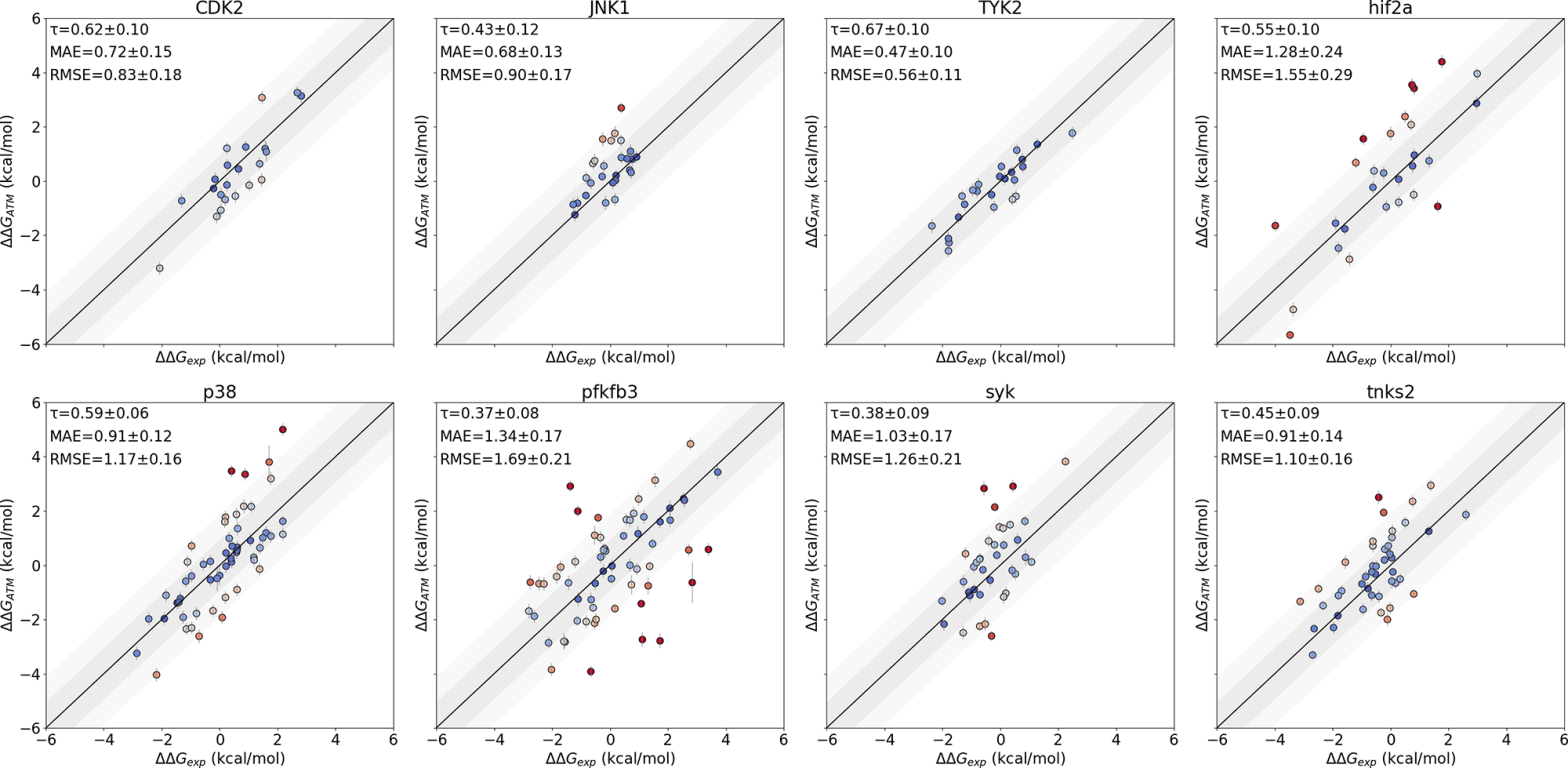
Performance in combination with the neural network potential (NNP) for each protein–ligand system studied. The calculated ΔΔ*G* estimates are plotted against their corresponding experimental values. MAE and RMSE are in kcal/mol and τ is Kendall correlation.

**Table 1:** Comparison of Performance of Different Force Fields and NNP/MM^a^.

	GAFF2			NNP/MM ANI2x			FEP+			
	Kendall (τ)	MAE	RMSE	Kendall (τ)	MAE	RMSE	Kendall (τ)	MAE	RMSE	ligand pairs
CDK2	0.42 ± 0.17	0.8 ± 0.2	1.1 ± 0.3	**0.62** ± **0.10**	**0.7** ± **0.1**	**0.8** ± **0.2**	0.42 ± 0.15	0.8 ± 0.1	1.1 ± 0.1	22
JNK1	0.34 ± 0.14	0.9 ± 0.2	1.0 ± 0.2	**0.43** ± **0.12**	**0.7** ± **0.1**	**0.9** ± **0.2**	0.43 ± 0.14	0.8 ± 0.1	1.0 ± 0.1	27
P38	0.48 ± 0.06	1.2 ± 0.2	1.6 ± 0.2	0.59 ± 0.05	0.9 ± 0.1	1.2 ± 0.2	**0.60** ± **0.11**	**0.8** ± **0.1**	**1.0** ± **0.1**	56
TYK2	0.33 ± 0.15	1.1 ± 0.2	1.3 ± 0.3	**0.67** ± **0.10**	**0.5** ± **0.1**	**0.6** ± **0.1**	0.54 ± 0.15	0.8 ± 0.2	0.9 ± 0.1	24
HIF2A	0.43 ± 0.10	1.6 ± 0.3	2.0 ± 0.4	**0.55** ± **0.11**	1.3 ± 0.2	1.6 ± 0.3	0.50 ± 0.13	**1.1** ± **0.1**	**1.3** ± **0.2**	28
PFKFB3	0.49 ± 0.06	1.3 ± 0.2	1.6 ± 0.2	0.37 ± 0.08	1.3 ± 0.2	1.7 ± 0.2	**0.70** ± **0.09**	**1.0** ± **0.1**	**1.6** ± **0.2**	62
SYK	0.25 ± 0.12	1.3 ± 0.2	1.6 ± 0.3	**0.38** ± **0.10**	**1.0** ± **0.2**	**1.3** ± **0.2**	0.16 ± 0.11	1.2 ± 0.1	1.5 ± 0.2	37
TNKS2	0.37 ± 0.09	1.0 ± 0.2	1.2 ± 0.2	**0.45** ± **0.10**	0.9 ± 0.1	**1.1** ± **0.2**	0.41 ± 0.10	**0.8** ± **0.1**	1.0 ± 0.1	45

a Kendall correlation (τ), mean absolute error (MAE), and root mean square error (RMSE) in kcal/mol for the eight tested Protein Targets. FEP+ is included as a state-of-the-art comparison.

## 4 Conclusion

We conducted relative binding free energy (RBFE) calculations using an innovative NNP/MM approach. Our findings demonstrate the substantial accuracy enhancement achieved by using an NNP/MM approach at the cost of increased computational time. Compared to conventional ligand force fields like GAFF2, the NNP/MM approach exhibited reduced mean absolute errors, with most systems reaching below 1 kcal/mol. However, we acknowledge that the current NNP used in this study is limited to neutral molecules and a limited set of elements, posing a constraint on our exploration of the vast chemical space. Future endeavors should focus on expanding the applicability of NNPs to include charged ligands, thereby broadening the scope of our investigations. An increase in computing performance is also needed, probably with the inclusion of other integrators that allow for higher timesteps. Due to limited computational resources, a random subset of all the possible calculations was computed. Although a potential bias could be included due to the nature of the subset, we believe to have sampled an extensive number of data points to understand the capabilities of RBFE along with the NNP/MM approach. Our work highlights the potential of NNP/MM for accurate RBFE calculations and underscores the importance of further advancing NNPs to encompass a broader range of molecular species and further improve the accuracy of these calculations.

## Supplementary Material



## Data Availability

The calculated free energy values and ligand and protein structures, as well as preparation scripts, are available at https://github.com/compsciencelab/ATM_benchmark/tree/main/ATM_With_NNPs

## Notes

Description of the workflow used for this work. Barplots of the pearson correlation and RMSE from the calculated versus experimental ΔΔ*G* values for all systems and compared methods. Scatterplots for the calculated Δ*G* on all the connected systems and the comparison between the compared methods as well as barplots from the corresponding MAE, RMSE errors, and R2 and spearman correlations. Barplots with the analysis of different speed performances between ATM and ATM combined with NNP/MM method. Scatterplots for a series of targets studied at different timesteps. Convergence analaysis based on the estimation of the ΔΔ*G* through simulated time. (PDF)
