# Supplementary material for: Enhancing Protein–Ligand Binding Affinity Predictions Using Neural Network Potentials
Source: J Chem Inf Model. 2024 Feb 20;64(5):1481–5. doi: 10.1021/acs.jcim.3c02031 (PMC11214867; doi:10.1021/acs.jcim.3c02031)
Supplement: Supplementary file 1 [file ci3c02031_si_001.pdf]

Supporting Information
Enhancing Protein-Ligand Binding Affinity Predictions using Neural Network Potentials

Francesc Sabanés Zariquiey,[†,‡] Raimondas Galvelis,[†,‡] Emilio Gallicchio,[¶] John D. Chodera,[§] Thomas E. Markland,[‖] and Gianni De Fabritiis[*,†,‡,⊥]

†Computational Science Laboratory, Universitat Pompeu Fabra, Barcelona Biomedical Research Park (PRBB), C Dr. Aiguader 88, 08003, Barcelona, Spain
‡Acellera Labs, C Dr Trueta 183, 08005, Barcelona, Spain
¶Department of Chemistry, Brooklyn College of the City University of New York; PhD Program in Chemistry, Graduate Center of the City University of New York; PhD Program in Biochemistry, Graduate Center of the City University of New York, NY 11210, USA
§Computational and Systems Biology Program, Sloan Kettering Institute, Memorial Sloan Kettering Cancer Center, New York, NY 10065, USA
‖Department of Chemistry, Stanford University, 337 Campus Drive, Stanford, CA, 94305, USA
⊥Institució Catalana de Recerca i Estudis Avançats (ICREA), Passeig Lluis Companys 23, 08010 Barcelona, Spain

E-mail: g.defabritiis@gmail.com

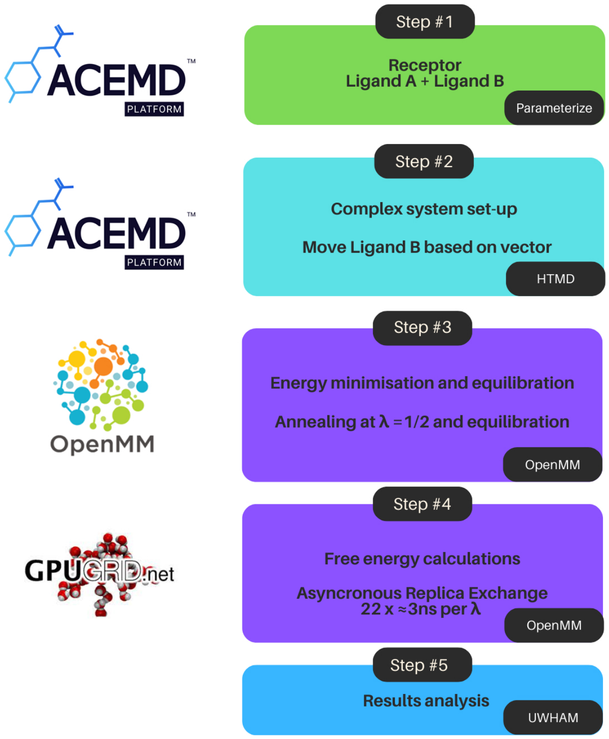

Figure S1: The ATM workflow used in this work. Ligands topologies are calculated with *parameterize* with GAFF2 and Sage force fields. (2) System complexes are prepared and built with htmd[?] . Protein topologies are prepared with the Amber ff14SB force field. Next ligand B is displaced based on a vector. (3) Energy minimization and equilibration is performed. Later an annealing and equilibration at λ=1/2 is performed. (4) Replica Exchange simulations are performed for a total sampling of 60ns. ATM simulations were run in GPUGRID were as ATM-NNP calculations were performed in our local cluster.(5) After the simulations were finished, these were analyzed with the UWHAM package to obtain the calculated $\Delta\Delta G$ estimates.

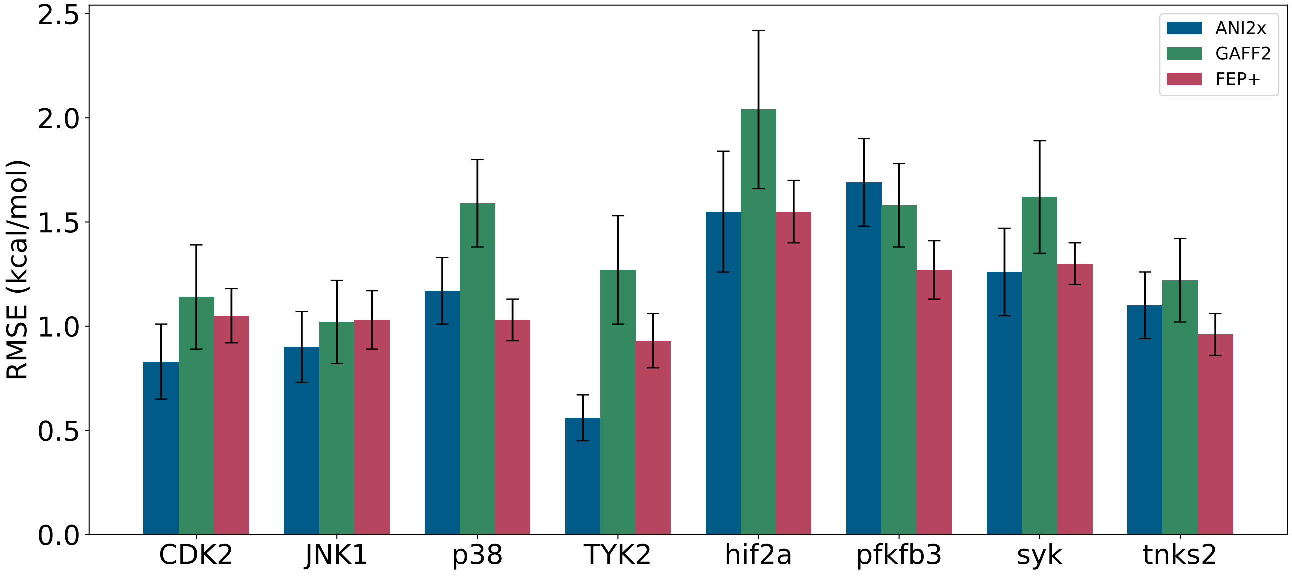

Figure S2: Pearson correlation for each protein-ligand system calculated in combination with different force fields and reported estimates using FEP+

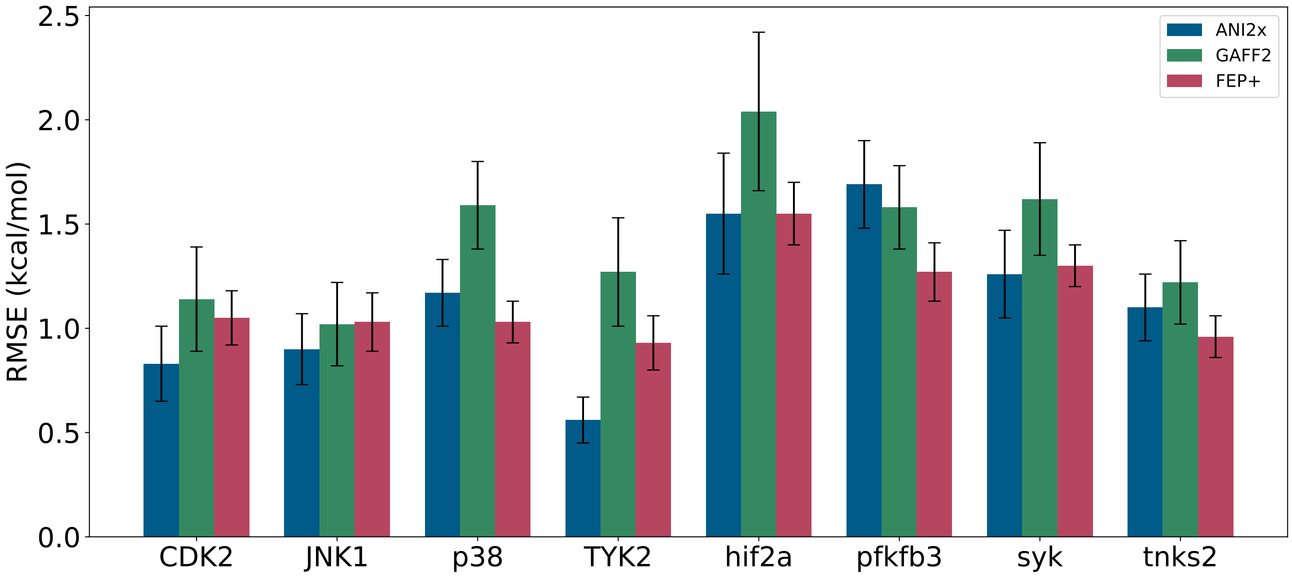

Figure S3: Root Mean Square Error (RMSE) in kcal/mol for each protein-ligand system calculated in combination with different force fields and reported estimates using FEP+

Table S1: Percentage of preditcions that have a MAE lower than 1 or 1.5 kcal/mol for each system.

**Protein**	**GAFF2**		**NNP/MM**		**FEP+**	
	**% MAE < 1**	**% MAE < 1.5**	**% MAE < 1**	**% MAE < 1.5**	**% MAE < 1**	**% MAE < 1.5**
**CDK2**	54.6 ± 4.9	71.1 ± 4.5	64.0 ± 9.3	96.0 ± 3.9	54.5 ± 10.3	86.4 ± 7.2
**JNK1**	54.9 ± 4.7	79.6 ± 3.7	74.1 ± 8.3	88.9 ± 6.0	70.4 ± 8.9	85.2 ± 6.8
**p38**	49.9 ± 2.5	65.4 ± 2.4	55.9 ± 6.2	81.4 ± 5.0	64.3 ± 6.3	83.9 ± 4.8
**TYK2**	48.0 ± 3.6	68.4 ± 3.3	85.0 ± 5.6	97.5 ± 2.5	87.5 ± 7.9	87.5 ± 7.9
**hif2a**	36.0 ± 4.6	55.9 ± 4.9	41.9 ± 8.6	58.1 ± 8.8	55.2 ± 8.9	75.9 ± 8.1
**pfkfb3**	41.8 ± 3.6	60.4 ± 3.6	42.9 ± 6.2	65.1 ± 6.2	61.3 ± 6.1	80.6 ± 5.1
**syk**	40.4 ± 4.6	61.4 ± 4.6	59.5 ± 8.0	78.4 ± 6.8	42.1 ± 8.0	73.7 ± 7.1
**tnks2**	55.8 ± 5.2	70.5 ± 4.7	66.7 ± 7.1	77.8 ± 6.3	75.6 ± 6.2	88.9 ± 4.2

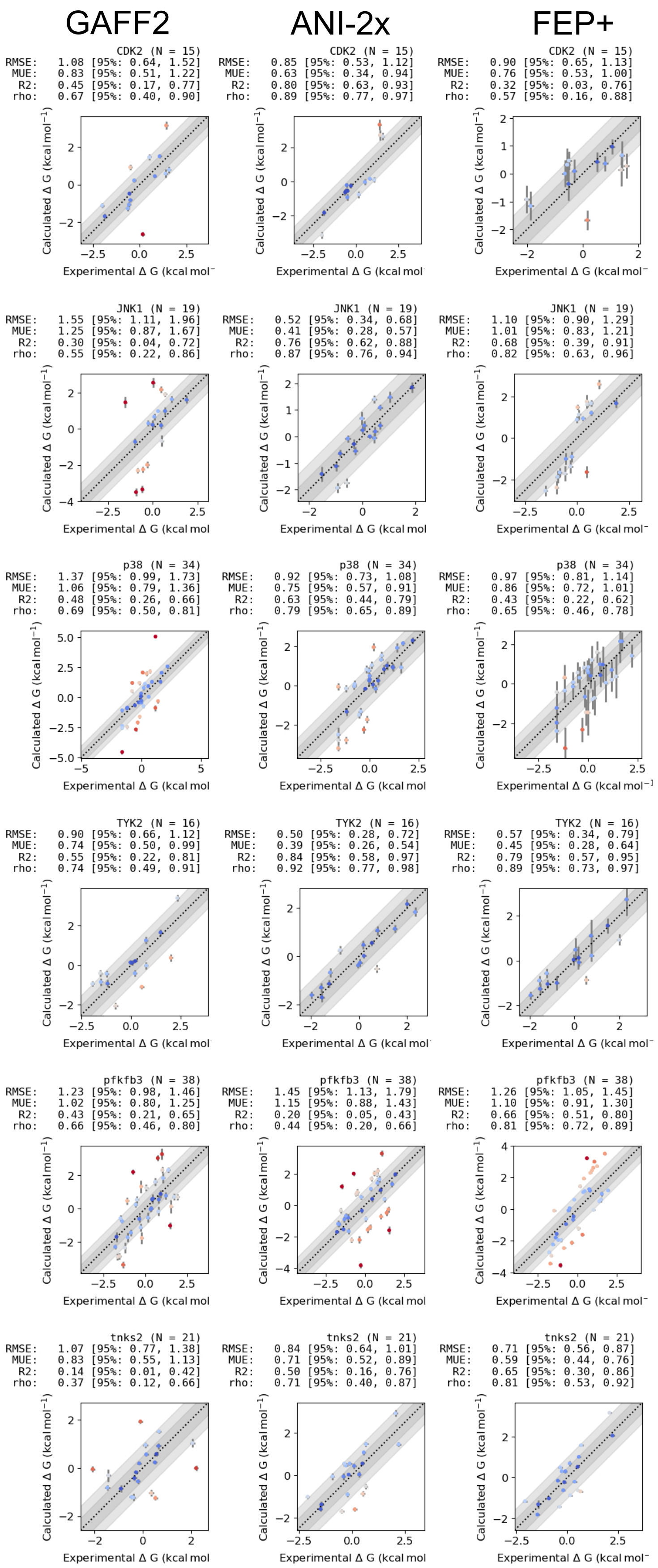

Figure S4: Scatterplots for the ΔG calculated on all the connected systems. Comparison between GAFF2, NNP/MM and FEP+. On top of each plot are the corresponding statistics.

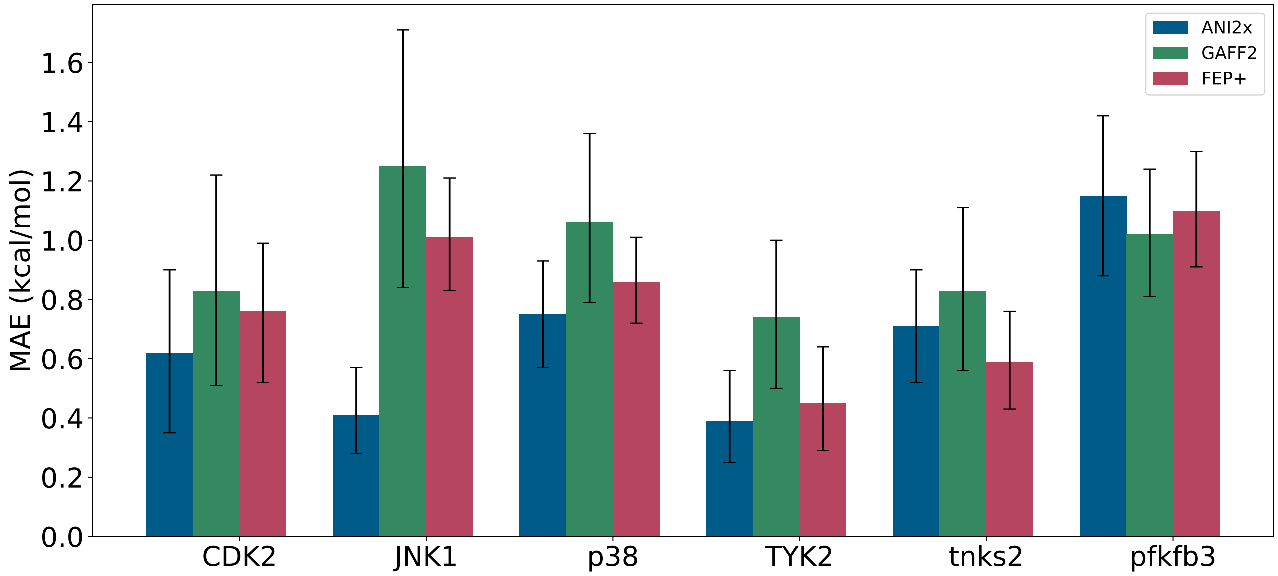

Figure S5: MAE (kcal/mol) for the ΔG values on all the connected systems

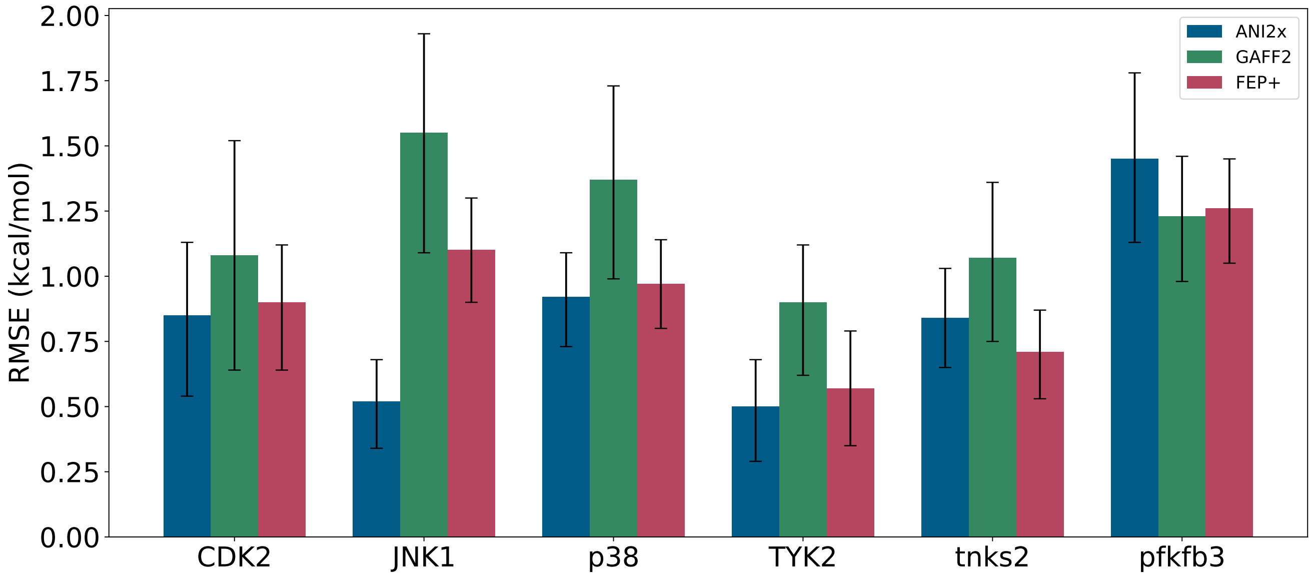

Figure S6: RMSE (kcal/mol) for the ΔG values on all the connected systems

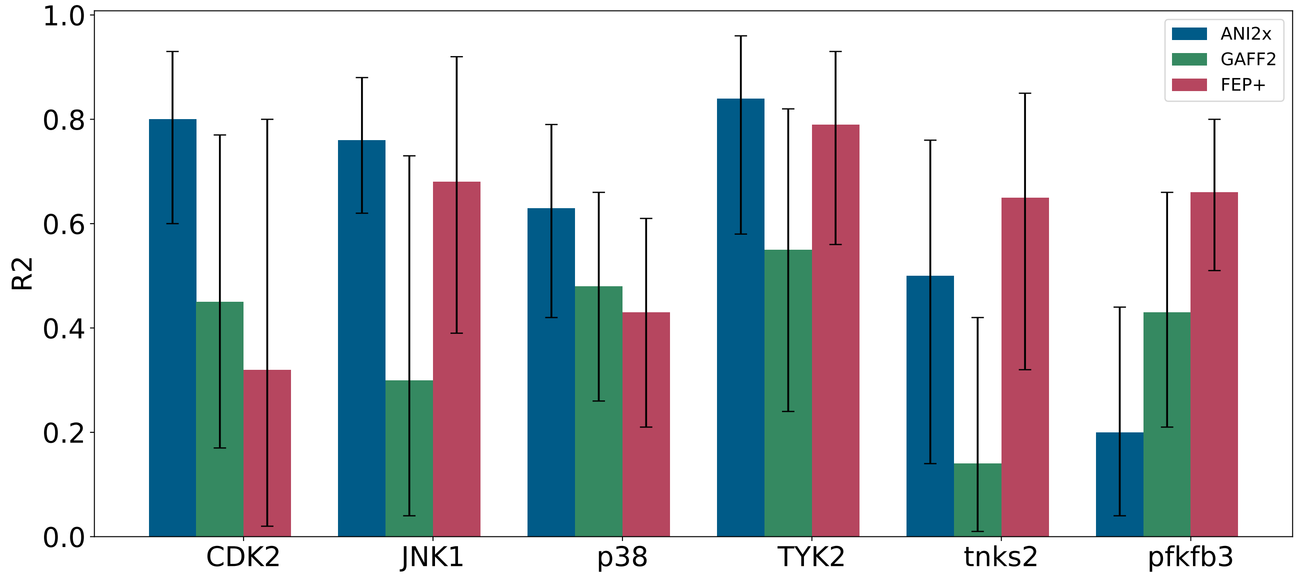

Figure S7: R2 correlation for the ΔG values on all the connected systems

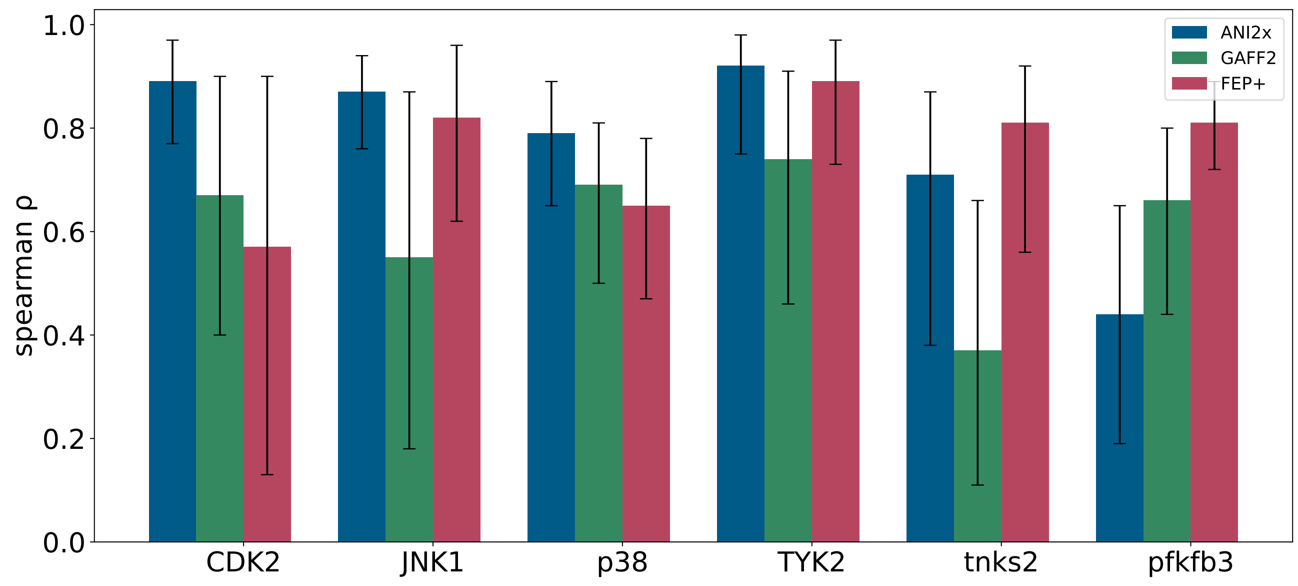

Figure S8: Spearman correlation for the ΔG values on all the connected systems

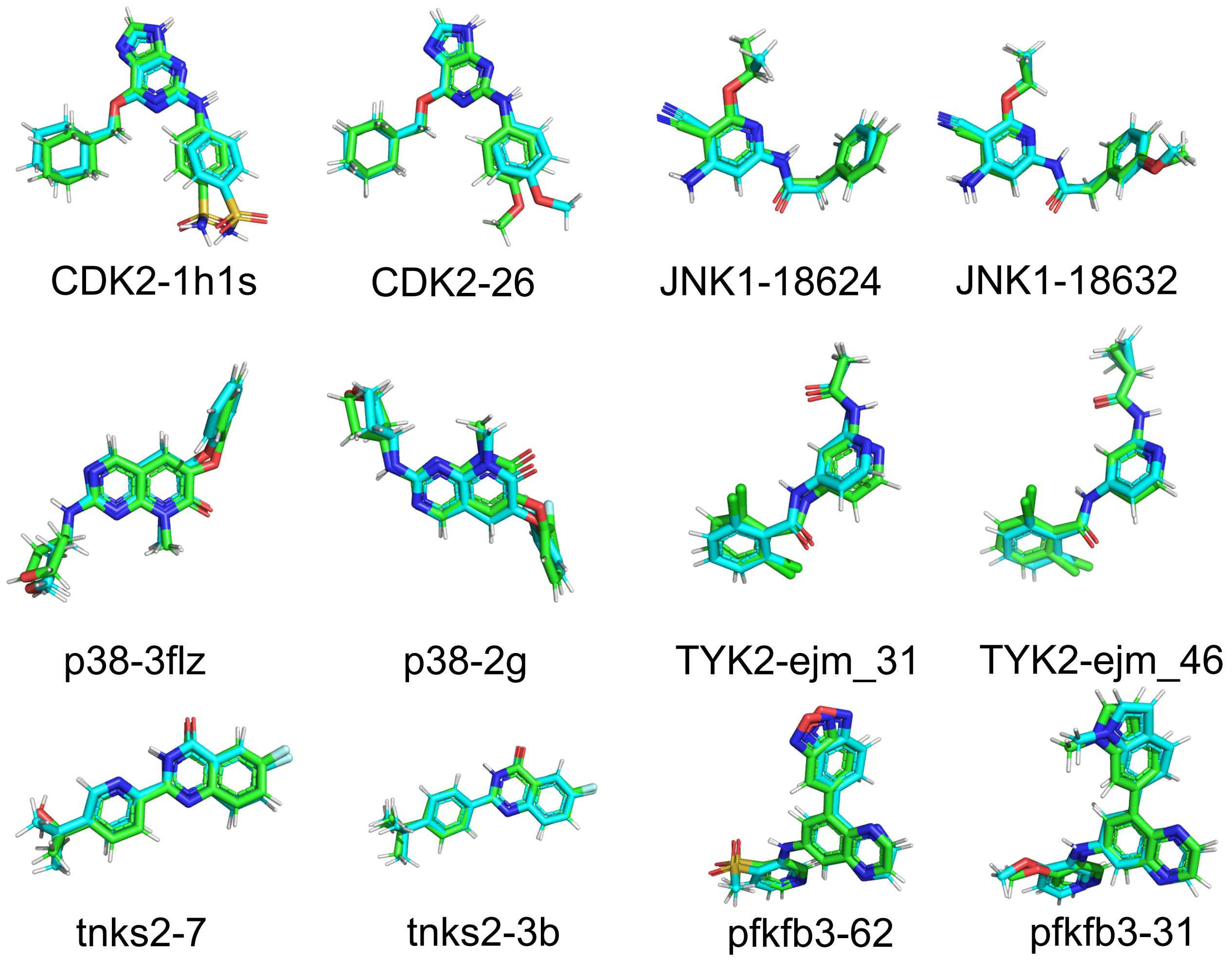

Figure S9: Generated conformers after equilibration for runs performed with GAFF2 (cyan) and ANI-2x (green).

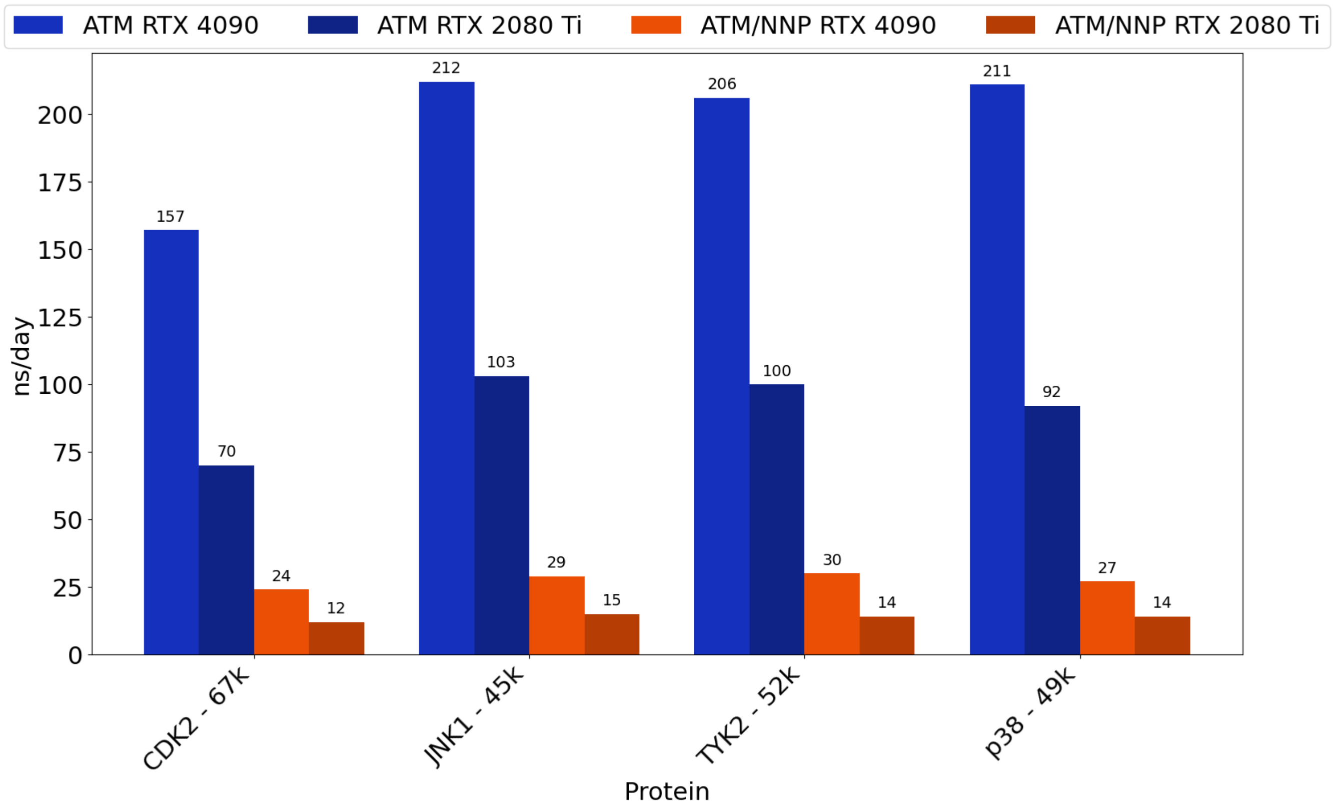

Figure S10: Performance of ATM and ATM/NNP on RTX 2080Ti and RTX 4090 graphics cards with OpenMM 7.7 MD engine and the ATM Meta Force plugin using the CUDA platform

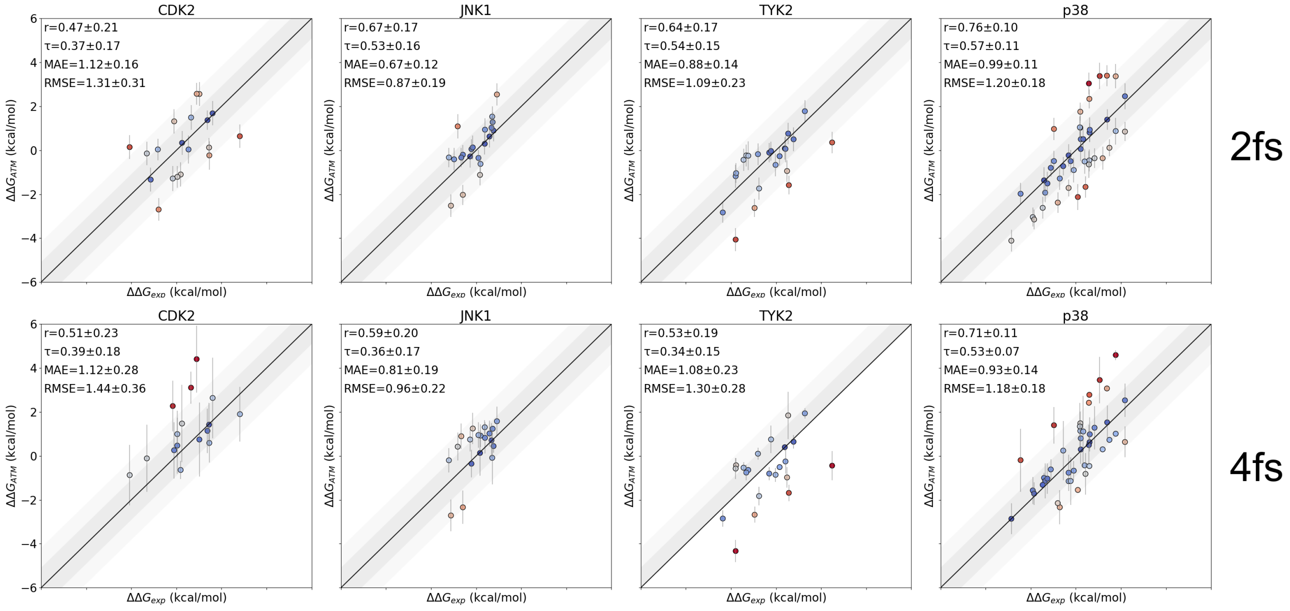

Figure S11: Scatterplots for a series of targets studied at different timesteps. Top row are the relevant ligand pairs studied in our previous work, which we realized with a 2fs timestep. Bottom row are the calculations done for these targets at a 4fs timestep.

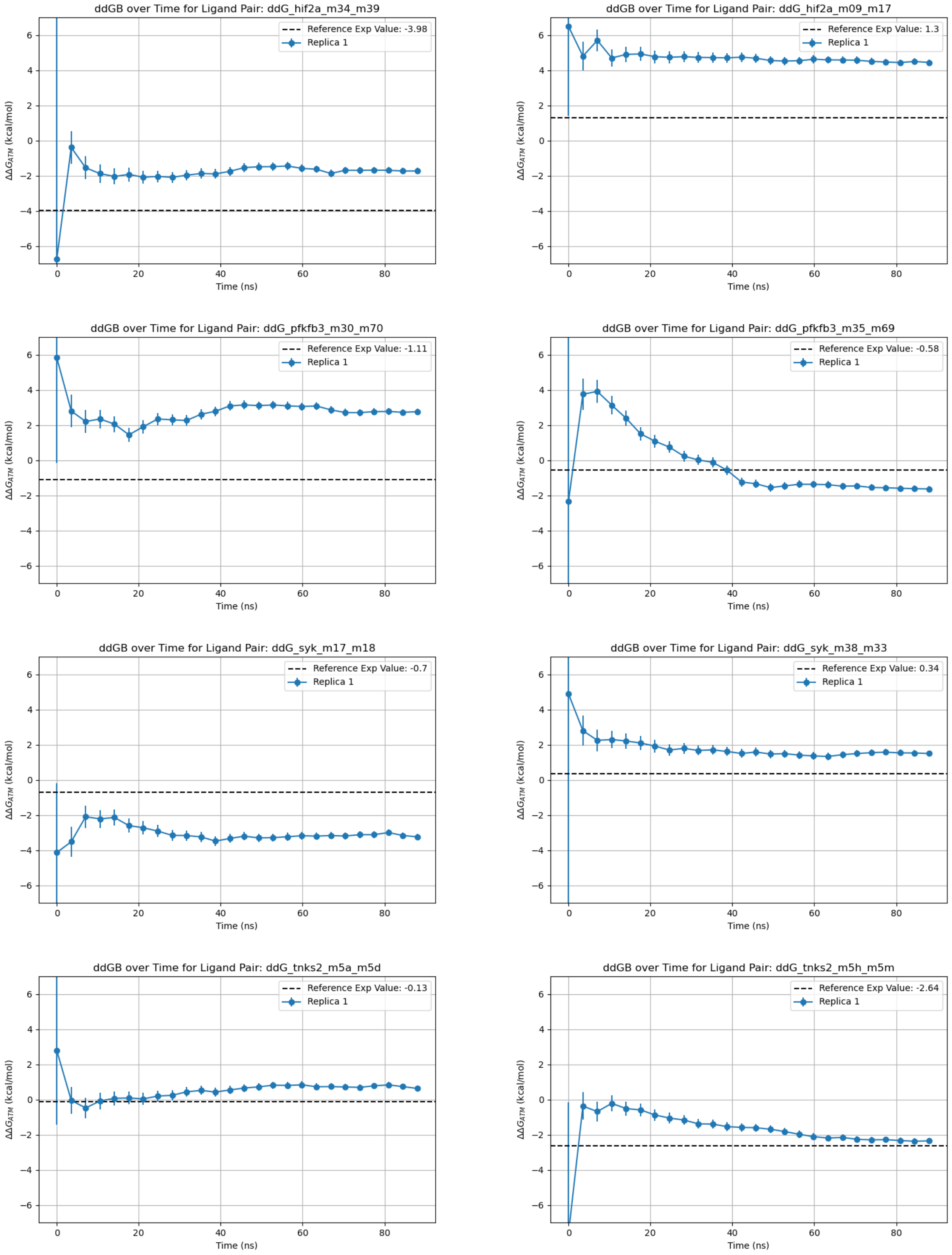

Figure S12: Free energy convergence as a function of time for a series of ligand pairs of hif2a, pfkfb3, syk and tnks2

Table S2: Case study example of the ΔΔGs obtained with NNP/MM and GAFF2. We observe how the transformations with the ligand ejm_ 55 give poor results with the GAFF2 (highlighted red) calculations but in the case of NNP/MM (highlighted green) the MAE is below 1kcal/mol.

Protein: TYK2			NNP/MM			GAFF2		
ligand1	ligand2	exp_ddG	ATM_ddG	error	MAE	ATM_ddG	error	MAE
ejm_31	ejm_46	-1.77	-2.27	0.25	0.50	-0.42	0.24	1.35
ejm_31	ejm_43	1.28	1.36	0.22	0.07	1.94	0.23	0.66
ejm_31	jmc_28	-1.44	-1.33	0.22	0.11	-0.54	0.23	0.90
ejm_31	ejm_45	-0.02	0.17	0.23	0.19	-0.86	0.23	0.84
ejm_31	ejm_48	0.54	-0.56	0.24	1.10	1.84	0.24	1.30
ejm_50	ejm_42	-0.80	-0.37	0.22	0.43	0.10	0.22	0.90
ejm_55	ejm_54	-1.32	-0.55	0.22	0.77	-0.76	0.23	0.56
ejm_43	ejm_55	-0.95	-0.33	0.23	**0.62**	-2.68	0.23	**1.73**
jmc_28	jmc_30	0.04	0.57	0.26	0.53	-1.07	0.28	1.11
jmc_28	jmc_27	-0.30	-0.50	0.22	0.20	-0.80	0.22	0.50
ejm_49	ejm_31	-1.79	-2.57	0.24	0.78	-0.57	0.24	1.22
ejm_49	ejm_50	-1.23	-0.86	0.24	0.38	-0.64	0.24	0.59
ejm_45	ejm_42	-0.22	-0.96	0.22	0.74	0.75	0.23	0.97
ejm_44	ejm_55	-1.79	-2.11	0.24	**0.32**	-4.33	0.23	**2.54**
ejm_44	ejm_42	-2.36	-1.65	0.27	0.71	-2.85	0.24	0.49
ejm_47	ejm_31	0.16	0.09	0.22	0.07	-0.51	0.23	0.67
ejm_47	ejm_55	0.49	0.04	0.22	0.44	-0.98	0.23	1.47
jmc_23	jmc_30	0.76	0.87	0.27	0.11	-0.25	0.25	1.01
jmc_23	ejm_46	0.39	0.33	0.22	0.06	0.40	0.22	0.01
jmc_23	ejm_55	2.49	1.77	0.23	**0.72**	-0.44	0.23	**2.93**
jmc_23	jmc_27	0.42	-0.67	0.24	1.09	-0.25	0.22	0.67
ejm_42	ejm_55	0.57	1.14	0.22	**0.57**	-1.68	0.22	**2.25**
ejm_42	ejm_48	0.78	0.53	0.22	0.25	0.64	0.23	0.14
ejm_42	ejm_54	-0.75	-0.12	0.22	0.62	-1.83	0.22	1.08

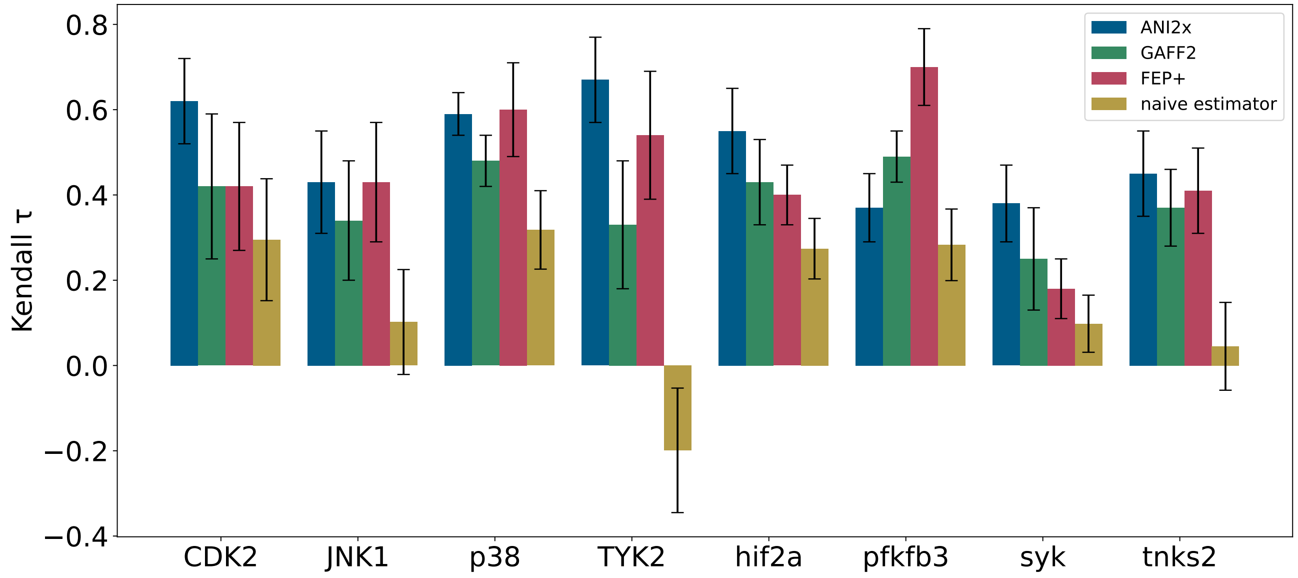

Figure S13: Comparison of Kendall tau for the $\Delta\Delta G$s of each protein-ligand system calculated and compared against a naive estimator based on the difference of molecular weight between ligands
